# Transcriptomics and quantitative proteomics reveal changes after second stimulation of bone marrow-derived macrophages from lupus-prone MRL/lpr mice

**DOI:** 10.3389/fimmu.2022.1004232

**Published:** 2022-10-19

**Authors:** Keyue Chen, Tiyun Wu, Danyan Wang, Rong Li, Xiangfeng Shen, Ting Zhao, Keiko Ozato, Rongqun Li

**Affiliations:** ^1^ Key Laboratory of Chinese Medicine Rheumatology of Zhejiang Province, School of Basic Medical Sciences, Zhejiang Chinese Medical University, Hangzhou, China; ^2^ Division of Developmental Biology, National Institute of Child Health and Human Development, National Institutes of Health, Bethesda, MD, United States

**Keywords:** systemic lupus erythematosus, innate immune memory, MRL/lpr mice, RNA-seq, proteome

## Abstract

Innate immune memory can cause the occurrence and exacerbation of autoimmune diseases, and it is as well as being strongly associated with the pathogenesis of systemic lupus erythematosus (SLE), however, the specific mechanism remains to be further studied. We learned that IFN-γ stimulation generated innate immune memory in bone marrow-derived macrophages (BMDMs) and activated memory interferon-stimulated genes (ISGs). This research used IFN-γ and lipopolysaccharide (LPS) to treat BMDMs with lupus-prone MRL/lpr mice and showed that particular memory ISGs were substantially elevated in prestimulated macrophages. In order to identify the differentially expressed genes (DEGs), researchers turned to RNA-seq. GO and KEGG analysis showed that up-regulated DEGs were enriched in defense and innate immune responses, and were related to the expression of pattern recognition receptors (PRRs)-related pathways in macrophages. TMT-based proteome analysis revealed differentially expressed proteins (DEPs) up-regulated in BMDMs were abundant in metabolic pathways such as glucose metabolism. Our study found that after the secondary stimulation of MRL/lpr mice, the expression of PRRs in innate immune cells was changed, and IFN-related pathways were activated to release a large number of ISGs to promote the secondary response. At the same time, related metabolic modes such as glycolysis were enhanced, and epigenetic changes may occur. Therefore, SLE is brought on, maintained, and worsened by a variety of factors that work together to produce innate immune memory.

## Introduction

Multiple autoantibodies are seen in Systemic Lupus Erythematosus (SLE) sufferers due to environmental variables such as recurrent virus infections and hormonal fluctuations, immune cells inappropriately multiply and activate to create a significant number of antibodies, which ultimately lead to numerous organ damage and illness (1). SLE has been related to a variety of viruses and bacteria, including the Epstein-Barr virus, human internal retroviruses, cytomegaloviruses, parvo B19, as well as human immunodeficiency type 1 (2). In the state of infection, viral/bacterial dsRNA/ssRNA or dsDNA can induce abnormal activation of the interferon (IFN) system, resulting in disturbance of immune regulation and response, leading to the onset of SLE (3, 4). Meanwhile, in SLE, increased apoptosis or suboptimal clearance leads to an increase in autoantigen-antibody complexes, which have been shown to be endogenous IFN inducers and can continue to produce IFN, forming a vicious circle (5). When the immune system is activated and fights viral infection, IFN is a crucial cytokine in the onset and progression of SLE (6–8). Extensive data have found that all childhood SLE and more than 65% of adult SLE are clearly associated with IFN, IFN-producing cells, and IFN-induced products (9, 10).

According to the literature, it was found that among the 2,000 IFN-β or IFN-γ stimulated genes (ISGs), more than 1,000 showed memory, called memory ISGs. Memory ISGs may show the histone H3.3 as well as H3K36me3 chromatin marks (11). Repeated external stimuli such as repeated viral infections can build innate immune memory in humans and mammals (12). Innate and acquired immunity make up the two main components of the immune system. Because acquired immunity contains immunological memory, it’s a significant distinction from innate immunity. Recent studies have found that after being stimulated by pathogenic microorganisms and their products, innate immune cells can exhibit non-specific immune-enhancing memory characteristics to the original stimulus when they encounter infection again, a phenomenon known as innate immune memory (13). Studies have found that there is a strong correlation between the innate immune memory and the incidence of certain disorders, including rheumatoid arthritis (14, 15), diabetes (16), and Alzheimer’s disease (17). At present, more and more scientific researches focus on the connection between immunological illness etiology and innate immune memory function. Innate immune memory is distinct from the classical adaptive immune memory, which depends on antigen-specific gene reshuffling, while the former relies on transcriptional regulators and epigenetic genome - editing signals (18). Unusually activated innate immune memory effects can cause excessive inflammatory processes that damage the body’s tissues. Anti-autoimmune illnesses may be caused by innate immune memory, according to several research. Notably, the metabolism of immune cells also changes in SLE patients, with possible epigenetic effects (19). At the same time, monocytes from SLE patients also undergo epigenetic reprogramming, and the surrounding histones in the TNF-α gene region are highly acetylated, so this gene is more easily transcribed (20), and histone H4 is highly acetylated (21). SLE-specific changes in the enhancers of H3K4me3 and H3K27me3 were also detected (22). Primary monocyte from SLE patients were subjected to genome-wide epigenetic analysis, and the results showed that H3K4me3 was substantially elevated in genes related to inflammation and the immunological response (23). SLE patients may have an innate immunological memory, as shown by these findings. We hypothesize that SLE’s etiology is intimately linked to the presence of memory ISGs.

Using PCR and gene chips, a DNA sequencing method, High-throughput sequencing, also known as next-generation sequencing (NGS), was developed as a new technology (24). Many complicated human disorders, such as autoimmune problems and cancer, have been studied using this technique (25). SLE-promoting and SLE-maintaining pathways may be elucidated by transcriptome analysis when the problem is handled at the cell type level. Cell-to-cell signaling reactions and cell-type specific pathways (24). A growing number of researchers are turning to proteomics as a supply of novel biomarkers for a variety of disorders. Quantitative proteomics methods based on tandem mass tags (TMT) have been extensively employed to date for protein biomarker research and the assessment of protein modification in several autoimmune diseases and malignancies (26). The activation and control of inflammatory responses, as well as tissue homeostasis, are all mediated by macrophages. Studies have shown that macrophages have a variety of roles in the onset of autoimmunity as well as the occurrence and progression of SLE (27). Signaling mechanisms that can start an inflammatory cascade and release inflammatory factors have been discovered in the bone marrow-derived macrophages (BMDMs) of mice (28). BMDM is frequently employed in experimental research on SLE. The BMDMs of MRL/lpr lupus mice were chosen as the research subject in our earlier study, and we discovered that stimulation of BMDMs by LPS elevated the high production of inflammatory cytokines and IRAK1-NF-kB inflammatory signaling pathway in BMDMs of MRL/lpr mice (29). In this experiment, We stimulated BMDMs of female lupus-prone MRL/lpr mice once and twice respectively. RNA sequencing (RNA-seq), TMT were used to screen for differentially expressed genes (DEGs), differentially expressed proteins (DEPs). And bioinformatics analysis was carried out on them, including identification analysis, expression difference analysis and function analysis.

This study intended to confirm the existence and expression of ISGs with innate immunological memory in MRL/lpr mice exceeds the expression level of the first time when stimulated for the second time. Gene/protein differences to determine the link between the pathogenesis of SLE and innate immune memory. Therefore, for diagnosis and therapy of the illness to have new targets and information.

## Materials and methods

### Mice

Female ICR mice and MRL/lpr mice that were 6 to 8 weeks old and in the specific pathogen free (SPF) status were chosen and acquired from Slac Laboratory Animal Co, Ltd. (Shanghai, China). These animals were kept in a barrier environment and daily light/dark cycles lasted for 12 hours. The mice were kept in an environment with a constant temperature of 25°C, a relative humidity range of 40–60%, and unrestricted access to a regular feed.

### BMDMs: Culture and treatment

Cells from the tibia and femur of mice were washed for the extraction of BMDMs. Cells were washed with pre-chilled phosphate buffered saline (PBS) and cultured in cell culture dishes 100 mm wide (Corning, New York City, USA). Dulbecco’s modified Medium consisting (DMEM) (Gibco, CA, USA) was supplemented with 15% FBS (Gemini, CA, USA), plus 25ng/ml macrophages colony pushing (M-CSF) (PeproTech, NJ, USA). To eliminate non-adherent cells, the cells were treated in 5 percent CO2 for four hours at 37°C before being rinsed with PBS. Fresh medium was then added to the cell suspension after 72 hours of incubation. Adherent BMDMs were employed for assays using 100 units/mL of murine synthetic IFN-γ (PeproTech, NJ, USA) as well as 1ug/ml of lipopolysaccharide (LPS) (PeproTech, NJ, USA) for the given durations of time after 120 hours of adhesion.

### Polymerase chain reaction quantitative real-time analysis

Total cellular RNA was extracted from BMDMs using Trizol (TAKARA) and then transformed into complementary DNA using the TAKARA Reverse Transcription System Kit (TAKARA, Dalian, China). cDNA was amplified with SYBR Premix Ex-Taq RT-PCR Kit (TAKARA). Quantitative real-time PCR amplification reaction was performed using a Roch LightCycler 96 SW1.1 equipment (Basel, Switzerland) (Rt-qPCR). The comparative Ct method (2^(-DDCt)) was used to analyze the data obtained. An internal control, GAPDH, was employed as a comparison. All nucleotide sequences of the primers used in the studies are shown in **Table 1**.

**Table 1:** Primer sequence.

Primer name	Sequence 5’-3’
IFIT3	Forward:GCTCAGGCTTACGTTGACAAGG Reverse: CTTTAGGCGTGTCCATCCTTCC
DDX58	Forward:AGCCAAGGATGTCTCCGAGGAA Reverse:ACACTGAGCACGCTTTGTGGAC
IRF7	Forward:CCTCTGCTTTCTAGTGATGCCG Reverse:CGTAAACACGGTCTTGCTCCTG

### RNA-seq

Trizol was used to extract total RNA, and Oligo (dT) magnetic beads were used to select for mRNA with associated protein structure in that RNA. Ion interruption was used to break the RNA into pieces of around 300bp. An enrichment step was performed after the collection was generated using PCR amplification, and a 450-bp library was chosen from among the fragments. One Agilent 2100 Bioanalyzer was used to verify the library’s quality, and libraries containing various Index sequences were proportionally combined based on the library’s effective concentration and the quantity of data it needed. A single-stranded library was created by diluting the pooled library to 2nM and denatured it with alkali. Then, using Next-Generation Sequencing (NGS), based on the Illumina sequencing platform, paired-end (PE) sequencing of these libraries was performed. The sample is sequenced on the computer to obtain an image file, which is converted by the software of the sequencing platform to generate the raw data of FASTQ, that is, the off-computer data. We use Cutadapt (v1.15) software to filter the sequencing data to get high quality sequence (Clean Data) for further analysis. The filtering criteria were: remove sequences with adapters at the 3’ end, and remove reads with an average quality score lower than Q20. The reference genome and gene annotation files were downloaded from genome website. This reference genome is: Mus_musculus.GRCm39.dna.toplevel.fa. The filtered reads were mapping to the reference genome using HISAT2 v2.0.5. HTSeq statistical comparison to the Read Count value of each gene, as the original expression of the gene, using Fragments Per Kilo bases per Million fragments (FPKM) to normalize the expression. Among the referenced transcriptomes, genes with FPKM > 1 are generally considered to be expressed.

### Analysis of differential expression genes

DEGs got screened using DESeq based on the following criteria: |log2FoldChange| > 1, P-value < 0.05, read count as input value for DESeq. The ggplots2 package in the R language was used to create volcano plots of gene expression profiles. Use the R language Pheatmap package to perform bidirectional clustering analysis on the union of differential genes and samples of all comparison groups, clustering according to the expression level of the same gene in different samples and the expression patterns of different genes in the same sample, using the Euclidean method to calculate the distance, the complete linkage is used for clustering.

### Analysis of gene ontology and pathway enrichment of DEG

Use topGO to perform GO enrichment analysis, calculate P-value by hypergeometric distribution method (the standard of significant enrichment is P-value <0.05), find out the GO terms with significantly enriched differential genes to determine the main biological functions performed by differential genes. ClusterProfiler (3.4.4) software was used to carry out the enrichment analysis of the KEGG pathway of differential genes, focusing on significantly enriched pathways with P-value < 0.05. According to the GO or KEGG enrichment results, the degree of enrichment is measured by Rich factor, false discovery rate (FDR) and the number of genes enriched in this pathway. Among them, Rich factor refers to the ratio of the number of enriched differential genes to the number of annotated genes in the GO Term/pathway. The greater the Rich factor, the greater the degree of enrichment. FDR generally ranges from 0 to 1, and the closer it is to zero, the more significant the enrichment is, and the results are shown in the bubble chart. Additionally, we chose the top 10 GO terms that had the most significant enrichment—i.e., the smallest p-value—and displayed the GO enrichment results in a bar graph.

### Protein-protein interaction network analysis

Search3 Tool for the Retrieval of Interacting Genes/Proteins (STRING) is a database of protein interactions produced by the European Molecular Biology Laboratory (EMBL), which contains the most powerful experimental evidence, data mining, and homology prediction of protein interactions. We performed protein interaction analysis based on the STRING database to reveal the relationship between target genes. When the PPI information of this species was included in the STRING database, we screened the PPI-action pairs with DEGs and Score>0.95 in the direct database on the basis of the findings of gene expression analyses. Then the relationship between all target genes was obtained, and Cytoscape was used to make a map. Finally, the nodes with higher degrees of interaction were considered as candidate genes.

### Analysis of differentially expressed proteins

SDT buffer was used for sample lysis and protein extraction. The TMT reagent was used to label 100 g of each sample’s peptide combination, as directed by the manufacturer (Thermo Scientific). Labeled peptides were fractionated by the High pH Reverse Phase Peptide Fractionation Kit (Thermo Scientific). LC-MS/MS analysis was performed on a Q Exactive mass spectrometer (Thermo Scientific) coupled to Easy nLC (Proxeon Biosystems, now Thermo Fisher Scientific). A database model called Decoy was utilized to calculate the FDR, and the basic requirements for credible peptides were Peptide FDR 0.01. To arrive at the protein ratios, we use the median value for all of the protein’s distinct peptides. All peptide ratios are normalized to the average protein ratio. After adjustment, the median peptide ratio should be 1. In the significant difference protein screening, Fold Change (FC) > 1.2-fold (more than 1.2-fold up-regulation or less than 0.83-fold down-regulation) and P value < 0.05 (T-test or other) were used as criteria.

### Bioinformatics analysis

The target protein set’s quantitative information is first standardized to the (-1,1) range. The Complexheatmap R package (R Version 3.4) was used to classify the two dimensions of sample and protein expression at the same time (distance algorithm: Euclidean, connection method: Average linkage), and generate a hierarchical clustering heat map.Subcellular localization prediction was performed using the method of CELLO (http://cello.life.nctu.edu.tw/). Blast2GO was used to annotate target protein collections with GO annotations. KEGG Automated Annotation Server (KAAS) was used to execute KEGG pathway annotation on target protein datasets. Fisher’s exact test was used to compare the distribution of each GO classification or KEGG pathway in the target protein set and the overall protein set, and the target protein set was subjected to GO annotation or KEGG pathway annotation enrichment analysis. In all studies, p values < 0.05 were considered significant.

## Results

### Innate immune memory in MRL/lpr mice

**Figure 1:**
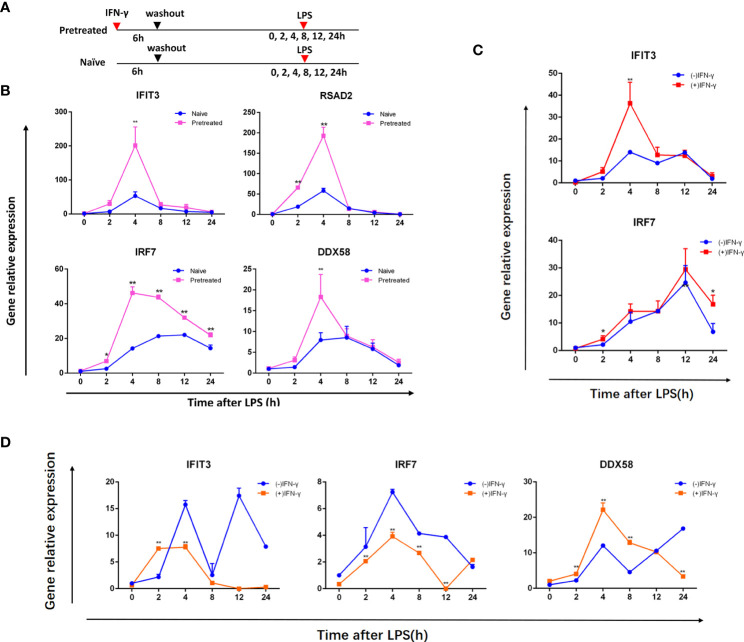
IFN stimulation produces memory in BMDMs from ICR mice and MRL/lpr lupus mice. **(A)** Treatment of cells. First, the naive and pretreated BMDMs were cultured for 24 hours lacking IFN after being treated with medium or 100 units/ml IFN over 6 hours, respectively. LPS (1ug/ml) was then added to these BMDMs and incubated for the given duration (in hours). qRT-PCR was used to assess ISG mRNA in BMDMs from 10-12 week old female MRL/lpr mice **(B)**, female ICR mice **(C)**, 16-18 week old female MRL/lpr mice **(D)**, which was normalized to Gapdh and represented as fold induction. (-) IFN-γ group was naive cells, (+) IFN-γ group was pretreated cells. Instances of memory ISGs are IFIT3, DDX58, IRF7 and RSAD2. The data represent the mean of three independent experiments ± SD. Statistically significant differences are indicated (Student’s t test, *P < 0.05, **P < 0.01).

MRL/lpr mouse is one of the most commonly used animal models of SLE. BMDMs from 10-12 week old female MRL/lpr mice were administered with IFN-γ (alluded to as IFN) for 6 hours, washed, and left minus IFN for 24 hours to examine whether or not they had innate immunological memory. Meanwhile, BMDMs cultured in normal medium without interferon were used as control group. Pretreated cells were then restimulated with LPS and IFN induction was compared to naïve cells previously not treated with IFN (shown in **Figure 1A**). Compared with naïve cells, the expression of IFIT3, DDX58, IRF7 and RSAD2 in pretreated cells increased faster and higher, and was most obvious at the 4th hour (shown in **Figure 1B**). In our earlier pilot studies, we initially chose 16-18 week old female MRL/lpr mice, but the PCR results revealed that the upward trend of ISGs, such as IFIT3, IRF7, DDX58, following the secondary stimulation was not significant (shown in **Figure 1D**). At the same time, our preliminary research demonstrated a trend toward higher ISG (IFIT3, IRF7) expression on the BMDM of ICR mice following secondary stimulation (**Figure 1C**), which was unrelated to the age of the mice.

### Identification of DEGs generated by BMDMs after primary versus secondary stimulation

**Figure 2:**
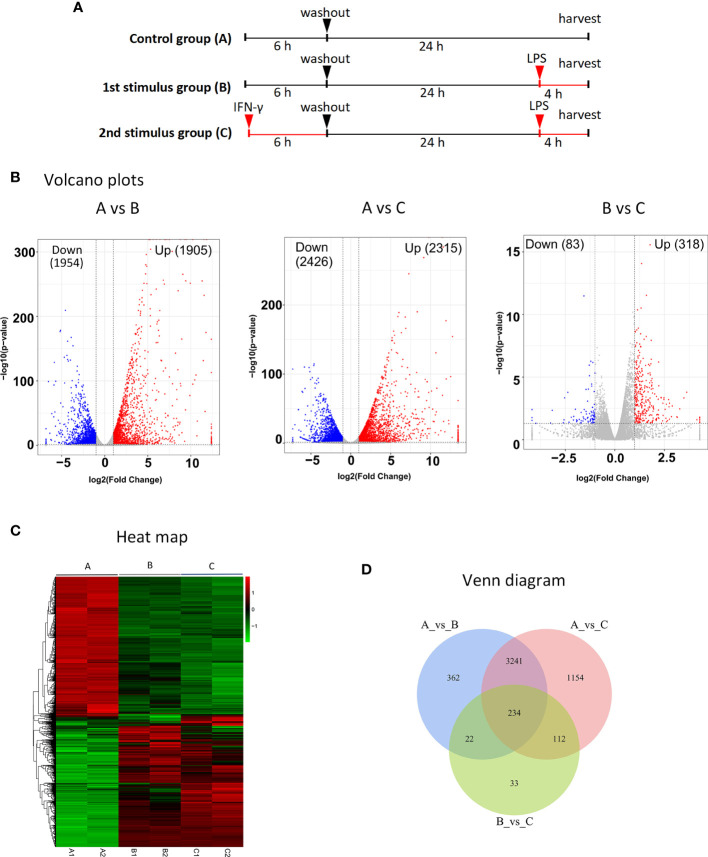
RNA-seq identification of DEGs after secondary stimulation of BMDMs in MRL/lpr mice **(A)** Experimental design for RNA-seq. The MRL/lpr mice’s BMDMs were divided into control group **(A)**, primary stimulation group **(B)** and secondary stimulation group **(C)**. Group A and group B were not treated, and group C was treated with IFN, washed after 6 hours, then incubated without IFN for 24 hours, and group B and group C were stimulated with LPS for 4 hours at the same time. DESeq was used for differential analysis of gene expression, and the conditions for screening DEGs were: |log2FoldChange| > 1, P-value<0.05. **(B)** Volcano plots of DEGs significantly up-regulated after secondary stimulation. Log2FoldChange is the abscissa and -log10 (p-value) is the ordinate. The horizontally dotted line shows the P-value=0.05 threshold, and the two vertical line(dotted) are the 2-fold interpretation difference thresholds in the image. In this group, red dots represent genes that are up-regulated, blue dots indicate genes that are down-regulated, and grey dots suggest genes that are not statistically different. **(C)** Clustering of different gene expression in six samples.The horizontal lines represent genes, each column is a sample, red represents high-expressed genes, and green represents low-expressed genes. Each group has two parallel samples, namely A1, A2, B1, B2, C1, C2. **(D)** Venn diagram of a total of 234 identical DEGs between the three datasets. Of these, 191 genes were up-regulated in all three datasets.

Three groups were formed: the control group (A), primary stimulus group (B), and secondary stimulus group (C) of MRL/lpr mice’s BMDMs. First, the treatment of groups A and B was not applied, and group C was treated with 100 units/ml IFN, washed after 6 hours, then incubated without IFN for 24 hours, and then stimulated group B and group C with LPS (1ug/ml) for 4 hours at the same time (shown in **Figure 2A**).

The volcano plots show the distribution of differential genes in each group, the magnitude of the gene expression fold change and the statistical significance found. Under normal conditions, the distribution of differential genes on the left and right sides of the graph should be roughly symmetrical, with the genes down-regulated by Case on the left and the genes up-regulated by Case on the right compared to Control. Compared with group B, group C after IFN pretreatment showed more than 300 DEGs, and most of them were up-regulated genes (shown in **Figure 2B**). Cluster analysis showed that there were differences in DEG among the three groups, and the DEGs in group A were significantly different from those in groups B and C (shown in **Figure 2C**). As demonstrated in Venn diagram (shown in **Figure 2D**), there were 234 DEGs across the three data sets, with 191 up- and 33 down-regulated genes in the study (shown in **Table 2**). By specifically analyzing DEGs in the BvsA and CvsA data, we discovered that, when compared to unstimulated BMDMs, there were certain common DEGs engaged in the process of histone methylation and acetylation at the time of primary and secondary stimulation, as shown in **Table 3**.

**Table 2:** Details of DEGs showing up or down regulation.

DEGs	Genes symbol
Up-regulated (191)	Gbp2, Gbp7, Il27,Tnfsf15, Il1rn, Cp, Gm6377, Ifitm3, Ctsc, Samsn1, Rapgef2, Ifih1, Slamf1, Hdc, Edn1, Gbp3, Abtb2, Herc6, Gbp2b, Slfn4, Calhm6, Atp10a, Zfp36, Zup1, Ddx60, Ifi44, Slc28a2, Fndc3a, Oasl1, Ddx58, Ms4a4c, Aida, Dck, Isg15, Dhx58, Ifit1, Trim30c, Cxcl10, H2-Q4, Trim30a, Ifi205, Ms4a4a, Zbp1, Lipg, Eif2ak2, Pcgf5, Il15, Sp140, Il15ra, Gm7592, Rnf213, Olfr56, Enpp4, Sp100, Pnpt1, Tmem176a, Gbp6, Ifi211, Ifi204, Creb5, Uba7, Ifi47, Setdb2, Irgm2, Gvin2, Oas3, Mmp25, Tap1, Usp18, Csrnp1, Irf7, Gm15433, Stat3, Igtp, Nlrc5, Tiparp, Oas1g, Phf11b, Ppm1k, AA467197, Etnk1, H2-Ab1, Slfn9, Tmem176b, Cxcl9, A630001G21Rik, C130026I21Rik, Gvin1, Batf2, Phf11d, Ifi202b, Ifi35, Marchf5, Daxx, Ifi44l, Mov10, Cst7, Plaat3, Ccl12, Nfxl1, Gbp4, Isg20, Nt5c3, H2-T22, Irgm1, Slamf7, Ifit2, Prpf38a, Tpst1, Rsad2, Il19, Cmpk2, Epsti1, Tgtp2, H2-T24, AC168977.1, Htra4, Heatr9, Cd86,Tasl, Ifit3, Mlkl, Zeb1, Scimp, Csprs, Sp110, Tmem67, Gm4951, Ccnd2, Iigp1, Ifi209, Pml, Trim30d, Tlr3, Ifi203, Parp11, Serpina3g, Slfn1, Ifit1bl1, Serpina3f, Ms4a6b, Stat1, Mab21l3, Dusp5, Sema4c, Tnfsf10, Phf11c, A530032D15Rik, Apol9b, Cass4, Gbp9, Sass6, Slc25a22, Ms4a4b, Slfn3, Dll1, Ido2, Ccr7, Il2ra, Tmem171, Nod1, Arid5a, Lrrc4, Crybg1, Ifit3b, Apol9a, Klrk1, Hat1, Samhd1, Slfn8, Nudt17, Gm5431, Ifi208, Melk, Phf11a, Phactr1, Slamf9, Tgtp1, Spats2l, Gm52955, Trp53i11, F830016B08Rik, Ifi214, AC132444.1, Gypc, Fgl2, Cysltr2, Igsf9, Gm4841, Pttg1, Ifnb1
Down-regulated (33)	Angptl2, Cebpa, Tmem273, Jade1, Tnfrsf21, Id1, Eef1aknmt, Zscan18, Pfkfb2, Fbxo21, Cyb5rl, Kank3, Shisa9, 3110082I17Rik, Gpr146, 9430015G10Rik, Klf9, Pold1, Dph7, Fbxo31, Klhl42, Pmm1, Zscan2, Sema6b, Rgs11, Fbxo10, Zfp248, Ccnf, Mphosph9, Etaa1, Mbd4, Cracr2b, Tle6

**Table 3:** Common DEGs involved in histone modifications after two stimulations in BMDMs of MRL/lpr mice.

	Name	Description
**up**	H3f3b	H3.3 histone B [Source:MGI Symbol;Acc:MGI:1101768]
	Hdac1	histone deacetylase 1 [Source:MGI Symbol;Acc:MGI:108086]
	Hat1	histone aminotransferase 1 [Source:MGI Symbol;Acc:MGI:96013]
	Mecp2	methyl CpG binding protein 2 [Source:MGI Symbol;Acc:MGI:99918]
	Setdb2	SET domain, bifurcated 2 [Source:MGI Symbol;Acc:MGI:2685139]
**down**	Hdac5	histone deacetylase 5 [Source:MGI Symbol;Acc:MGI:1333784]
	L3mbtl3	L3MBTL3 histone methyl-lysine binding protein [Source:MGI Symbol;Acc:MGI:2143628]
	H2az2	H2A.Z histone variant 2 [Source:MGI Symbol;Acc:MGI:1924855]
	H2bc21	H2B clustered histone 21 [Source:MGI Symbol;Acc:MGI:2448415]
	H1f2	H1.2 linker histone, cluster member [Source:MGI Symbol;Acc:MGI:1931526]
	Hdac6	histone deacetylase 6 [Source:MGI Symbol;Acc:MGI:1333752]
	Hdac10	histone deacetylase 10 [Source:MGI Symbol;Acc:MGI:2158340]

### Analyses of DEGs using gene ontology as well as Kyoto encyclopedia of genes and genomes

**Figure 3:**
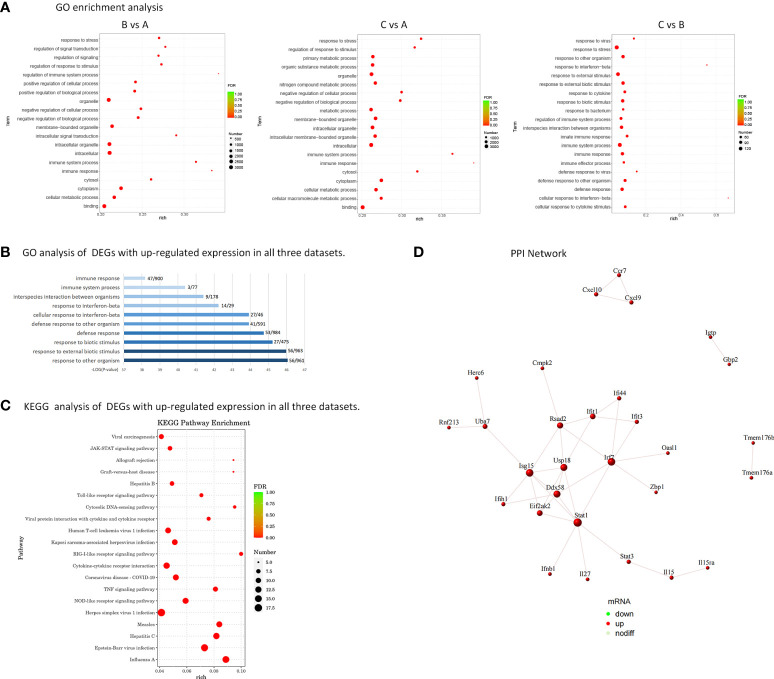
Biological analysis of DEGs. **(A)** GO enrichment analysis of DEGs. Rich factor refers to the ratio of the number of DEGs enriched in the GO term to the number of total genes annotated. The larger the rich factor, the greater the degree of enrichment. FDR generally ranges from 0 to 1, and the closer it is to zero, the more significant the enrichment is. The top 20 GO Term entries with the smallest FDR value, that is, the most significant enrichment, are selected for display. **(B)** Histogram of GO enrichment analysis. 191 DEGs with up-regulated expression in all three datasets were analyzed for GO enrichment. The top 10 GO term entries with the most significant enrichment in GO are selected for display. The abscissa is the terms of Go level2, and the ordinate is the -log10 (p-value) enriched for each term. The number of the genes identified in this study vs total gene numbers in the term are shown on the right. **(C)** Analysis of KEGG enrichment using a bubble plot. KEGG enrichment analysis of 191 DEGs with up-regulated expression. The top 20 KEGG pathways with the smallest FDR value are selected for display. **(D)** PPI Network - Schematic Diagram of Gene Expression. There are 12 key genes that are more closely related.

Comparing groups B and C with group A, the GO analysis of DEGs showed that they were enriched in intracellular, immune system process, regulation of biological and cellular process, response to stimulus. On the other hand, group C was compared with group B, GO analysis of DEGs showed that they were enriched in defense response, innate immune response, response to interferon−beta, response to external stimulus (shown in **Figure 3A**). Subsequently, biological analyses were performed on DEGs that showed up-regulation in all three datasets, that is, genes with memory. The top 10 GO term items with the smallest p-value, that is, the most significant enrichment, are selected for display. It can be seen that DEGs are significantly enriched in responses to external biological stimuli, responses to interferon-β, and immune system processes (shown in **Figure 3B**).

Displaying the top 20 KEGG routes with the least FDR value, which is to say the most meaningful enrichment, was done based on the findings of the KEGG enrichment study. The bubble plot (shown in **Figure 3C**) displays the findings. The majority of DEGs focus on disorders caused by viral infections, NOD-like receptor signaling pathway, TNF signaling pathway, RIG-I-like receptor signaling pathway, Toll-like receptor signaling pathway, JAK-STAT signaling pathway.

### Analysis of the PPI network

The protein interaction analysis of 191 up-regulated DEGs was performed according to the STRING database, and the maps were performed by Cytoscape (shown in **Figure 3D**). 22 genes are extremely closely linked in the PPI network: Irf7, Stat1, Rsad2, Eif2ak2, Usp18, Isg15, Ddx58, Ifit1, Uba7, Ifit3, Ifi44, Ifih1, Herc6, Rnf213, Ifnb1, Il27, Stat3, Il15, Il15ra, Zbp1, Oasl1, Cmpk2. Among them, the genes with more obvious PPI interactions are Irf7, Stat1, Rsad2, Eif2ak2, Usp18, Isg15, Ddx58, Ifit1, Uba7, Ifit3, Ifi44, Ifih1, which have nodes with high degree of interaction and can be used as key candidate genes, with innate immune memory, may be more critical in the pathogenesis of SLE. **Table 4** lists the genes and their roles for each of the individuals mentioned above.

**Table 4:** Key genes with innate immune memory in MRL/lpr mice.

Gene symbol	Full name	Details
Irf7	interferons regulatory factor7	Apoptosis, cancer, host defense, viral latency, and immunological response are a few of the processes regulated by this protein, which is mostly present in B - cell, plasmacytoid dcs (PDCs), and monocytes. It is also essential for type I IFN-mediated intrinsic immunity (30). Regulation of IRF7 expression and IRF7-mediated immune processes play important roles in SLE (31).
Stat1	signal transducer and activator of transcription 1	Transcription factors, signal transducers and transcription activators of the STAT family, play key roles in immune responses and IFN signaling pathways, regulating various cellular processes (32).
Rsad2	radical S-adenosyl methionine domain containing 2	Type I IFN-mediated viral inhibitory protein, widely involved in antiviral activity. Some studies have found that the expression of methylated RSAD2 in naive CD4+ T cells of SLE patients is significantly increased, and the methylation of the RSAD2 gene CpG site is related to the production of SLE-related autoantibodies (33).
Eif2ak2	eukaryotic translation initiation factor 2-alpha kinase 2	Also known as protein kinase R (PKR), responsible for the regulation of protein synthesis by phosphorylating the translation initiation factor eIF2α of serine-51, the eIF2α kinase PKR can regulate both global and specific mRNA translation in response to a variety of different stimuli, in particular in the immune response (34). EIF2AK2 selectively regulates immune responses and transcription of SLE-related histone genes (35).
Usp18	ubiquitin specific peptidase 18	Cellular activities such as signal transduction, stress responses and the response to pathogenic microbes all rely on this protein. SLE development may be aided by USP18 altering transcribed mRNA degradation and commencement of translation (36).
Isg15	ISG15 ubiquitin-like modifier	Ubiquitin-like proteins, either through their coupling to target proteins (ISGylation) or through their role as free or unbound proteins, changes the adaptive immune system to viral infection have a critical role (37).
Ddx58	DEAD/H box helicase 58	Also known as retinoic-acid-inducible gene I (RIG-I). A 925-residue cytoplasmic viral RNA receptor, also a member of the RIG-I-like receptor (RLR) family, is an essential intracellular sensor for several viruses that elicits antiviral IFN responses by recognizing viral double-stranded RNA (dsRNA), whose persistent abnormal activation can cause autoimmune diseases (38).
Ifit1	interferon-induced protein with tetratricopeptide repeats 1	Interferon-induced antiviral RNA-binding protein that specifically binds to single-stranded RNA containing a 5’-triphosphate group (PPP-RNA), thereby acting as a sensor for viral single-stranded RNA and inhibiting the expression of viral messenger RNA. IFIT1 is the first gene identified as a potential pathogenic factor for SLE (39).
Uba7	ubiquitin-like modifier activating enzyme 7	A specific E1-like ubiquitin-activating enzyme involved in ISG15 coupling and acts as an antiviral (40).
Ifit3	interferon-induced protein with tetratricopeptide repeats 3	IFN-induced antiviral protein that acts as an inhibitor of cellular and viral processes, cell migration, proliferation, signaling and viral replication. IFIT3 is one of the genes responsible for the overactive cGAS/STING signaling pathway in human SLE monocytes (41).
Ifi44	interferon-induced protein 44	It is an interferon-inducible gene involved in various biological effects of interferon signaling, such as antiviral. IFI44 is considered a key biomarker in lupus nephritis (LN) (42).
Ifih1	interferon induced with helicase C domain 1	Also known as melanoma differentiation-associated protein 5 (MDA5), the innate immune receptor, which acts as a cytoplasmic sensor of viral nucleic acids, plays a major role in sensing viral infection and activating a range of antiviral responses. IFIH1 may contribute to SLE pathogenesis by altering inflammatory mechanisms (43).

### qRT-PCR validation of the related genes

**Figure 4:**
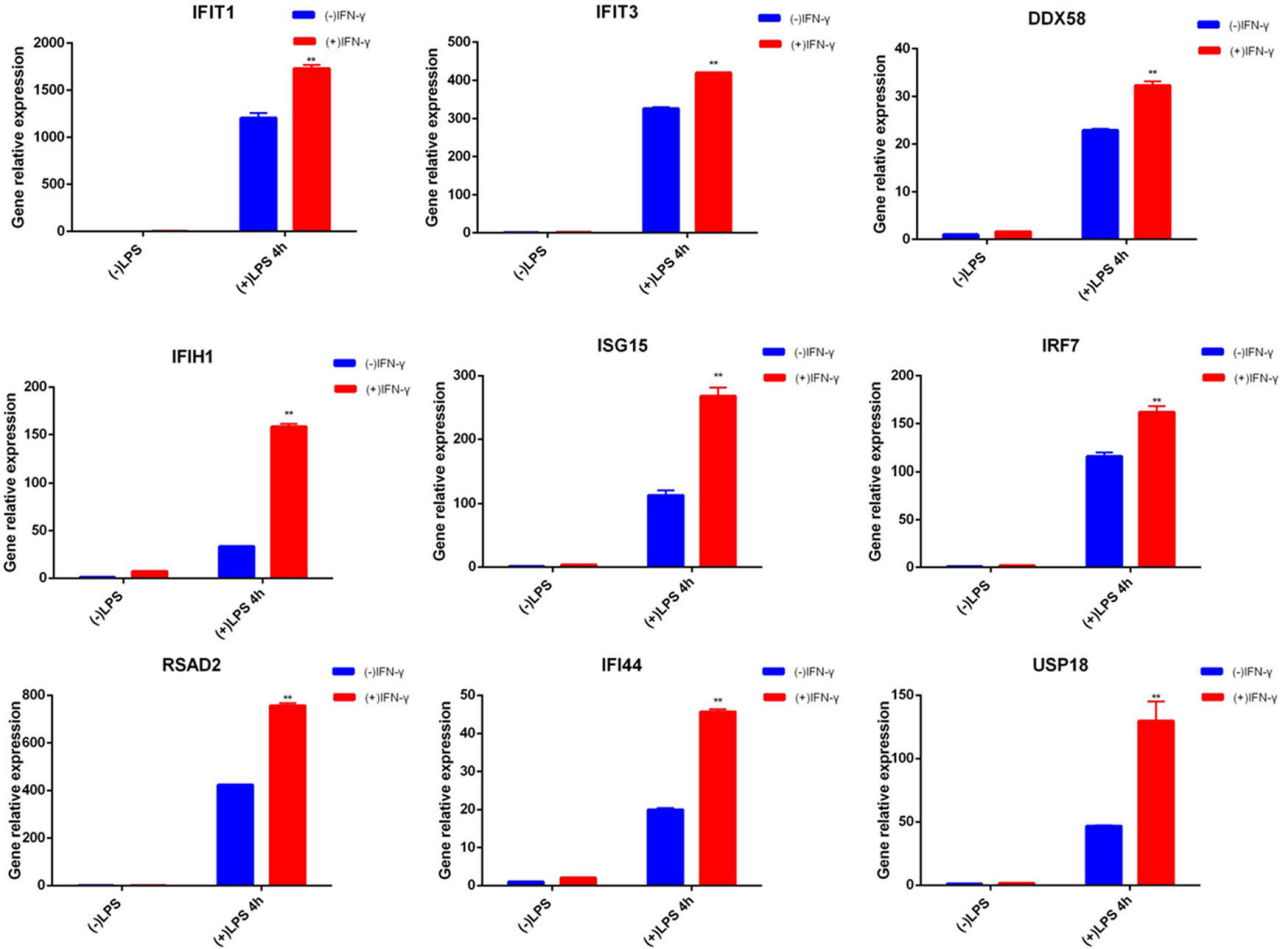
qRT-PCR of related genes. Blue represents cells not pretreated with IFN-γ, and red represents cells treated with IFN-γ for 6 hours. The two groups of cells were treated with LPS for 4 hours after cultured in normal medium for 24 hours. ISG mRNA was measured by qRT-PCR, normalized by Gapdh, and expressed as fold induction. Nine genes are examples of memory ISGs in the transcriptome, respectively. Data represent the mean ± SD of three independent experiments. Statistically significant differences are indicated (Student’s t test, **P < 0.01).

For the relevant gene validation with innate immune memory, we selected 9 key candidate genes (IFIT1, IFIT3, DDX58, IRF7, IFIH1, ISG15, RSAD2, IFI44, USP18). qRT-PCR was used to examine the corresponding mRNA expression levels of these selected genes (shown in **Figure 4**). qRT-PCR results for the selected genes were consistent with the results of the transcriptomic data.

### The protein changes in MRL/lpr mice BMDMs after primary stimulation and secondary stimulation

**Figure 5:**
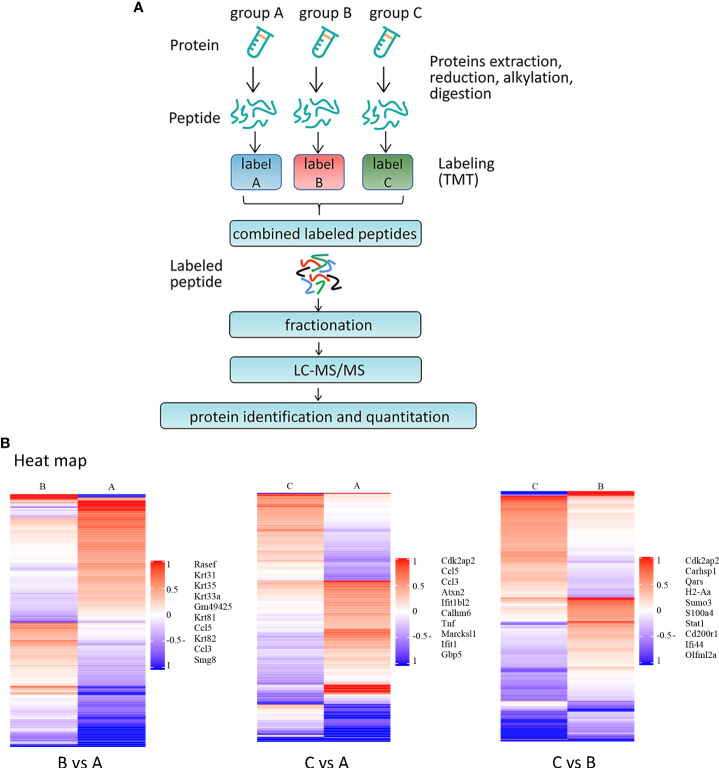
Proteomic analysis of BMDMs from MRL/lpr after primary stimulation and secondary stimulation **(A)** Schematic overview of TMT-based quantitative proteomic analysis of BMDMs samples from MRL/lpr mice. The grouping and culture of BMDMs are shown in **Figure 2A**. Samples were reduced, alkylated and digested with trypsin. Samples labelled with TMT were analysed by LC-MS/MS. **(B)** Results of cluster analysis of DEPs. The target protein set’s quantitative information is first standardized to the (-1,1) range. The Complexheatmap R package (R Version 3.4) was used to classify the two dimensions of sample and protein expression at the same time (distance algorithm: Euclidean, connection method: Average linkage). The tree-type heatmap represents the hierarchical clustering results. For each sample, the abscissa represents sample information, and for each row, it represents a protein; for this reason, an ordinate signifies a protein with substantially differing expression levels. Colors in the heatmap reflect up- and down-regulated proteins, respectively, based on the log2 normalization approach, where red represents significantly up-regulated proteins, blue represents significantly down-regulated proteins, and gray represents no protein quantitative information. Names of representative proteins are shown.

Next, we performed proteomic analysis (shown in **Figure 5A**). In order to analyze the expression patterns of samples between groups and within groups, test the rationality of the grouping of this project, and indicate whether the changes in differential protein expression can represent the significant impact of biological treatment on the samples, a hierarchical clustering algorithm was used to compare the groups. A heatmap is used to show the DEPs that have already been grouped and classed in a visual manner. DEPs screening may successfully differentiate comparison groups (shown in **Figure 5B**) by the screening criterion of fold change > 1.2 times and P < 0.05 (T-test or other), indicating that can represent the influence of biological treatment on samples.

DEPs across various groups were analyzed by screening the experimental data for differences. In the DEPs screening, the number of up-regulated and down-regulated proteins between the comparison groups was obtained by taking Fold Change (FC) > 1.2-fold (up-regulation more than 1.2-fold or down-regulation less than 0.83-fold) and P < 0.05 (T-test or other) as the standard. Compared with the control group, BMDMs after one stimulation showed 579 (298 up and 281 down) DEPs, and BMDMs after two stimulations showed 578 (279 up and 299 down) DEPs. Compared with the primary stimulation group, the secondary stimulation group exhibited 445 (201 up and 244 down) DEPs (shown in **Table 5**). In addition, among the three datasets, there were 10 DEPs that are all up-regulated and 20 DEPs that are all down-regulated. **Table 6** lists the precise names of them.

**Table 5:** Statistical table of protein quantitative difference results (A: Control group; B: Primary stimulation group; C: Secondary stimulation group).

Comparisons	Significantly changing in abundance		
	Upregulated	Downregulated	All
B_vs_A	298	281	579
C_vs_A	279	299	578
C_vs_B	201	244	445

**Table 6:** Details of DEPs that were up- or down-regulated in all three datasets.

	Protein name	Gene name
Up-regulated (10)	CDK2-associated protein 2	Cdk2ap2
	interferon induced protein with tetratricopeptide repeats 1B like 2	Ifit1bl2
	calcium homeostasis modulator family member 6	Calhm6
	ISG15 ubiquitin-like modifier	Isg15
	guanylate binding protein 2	Gbp2
	interferon inducible GTPase 1	Iigp1
	interferon gamma inducible protein 47	Ifi47
	CD74 antigen (invariant polypeptide of major histocompatibility complex, class II antigen-associated)	Cd74
	glutamate rich 1	Erich1
	ubiquitin-conjugating enzyme E2I	Ube2i
Down-regulated (20)	inner membrane protein, mitochondrial	Immt
	heterogeneous nuclear ribonucleoprotein D	Hnrnpd
	LIM and SH3 protein 1	Lasp1
	thymopoietin	Tmpo
	serine/arginine repetitive matrix 1	Srrm1
	Sad1 and UNC84 domain containing 1	Sun1
	fibrinogen gamma chain	Fgg
	advanced glycosylation end product-specific receptor	Ager
	hemoglobin alpha, adult chain 1	Hba-a1
	hemicentin 1	Hmcn1
	SAM domain and HD domain, 1	Samhd1
	small nuclear RNA activating complex, polypeptide 4	Snapc4
	SFT2 domain containing 2	Sft2d2
	centrosomal protein 162	Cep162
	ubiquilin 4	Ubqln4
	glutaredoxin 2 (thioltransferase)	Glrx2
	INTS3 and NABP interacting protein	Inip
	polymerase (RNA) II (DNA directed) polypeptide I	Polr2i
	stearoyl-coenzyme A desaturase 3	Scd3
	phospholipase C, beta 4	Plcb4

### Biological analysis of DEPs between different groups

**Figure 6:**
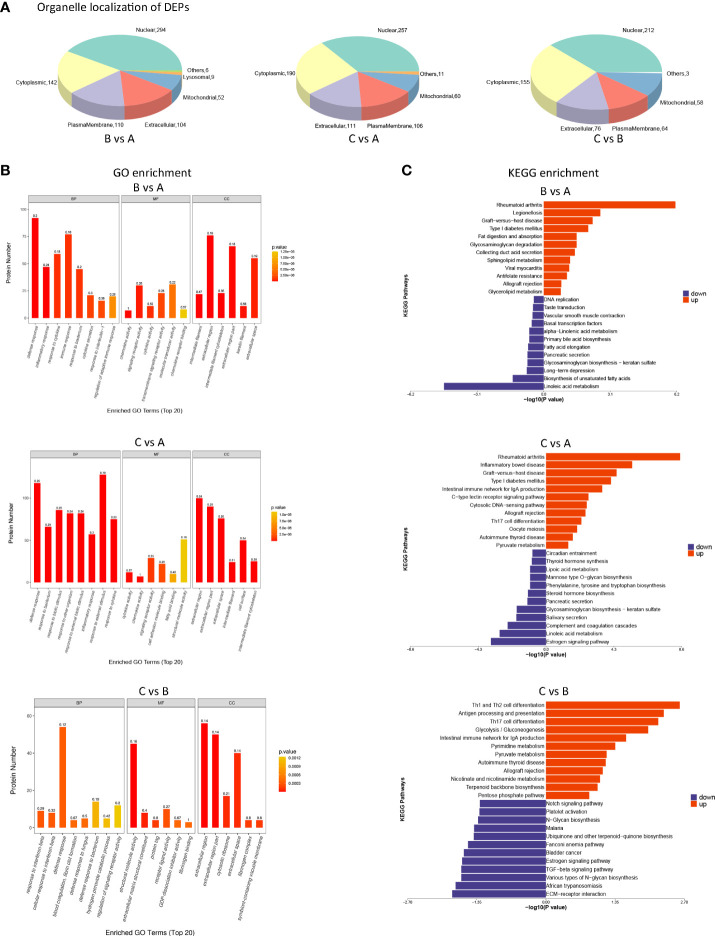
Biological analysis of differentially expressed proteins (DEPs) between different groups **(A)** Pie chart of organelle localization of DEPs. eSubcellular localization prediction was performed using the method of CELLO (http://cello.life.nctu.edu.tw/). At the same time, the number and distribution ratio of proteins in each subcellular organelle are displayed in the form of a pie chart. **(B)** GO enrichment analysis of DEPs. Blast2GO was used to annotate target protein collections with GO annotations. GO enrichment findings of DEPs were classified as molecular function (MF), biological process (BP), and cellular component (CC). **(C)** KEGG pathway annotation statistics of DEPs (Top20). KEGG Automated Annotation Server (KAAS) was used to execute KEGG pathway annotation on target protein datasets.

Organelles are functionally independent subcellular units in a cell, each of which has a specific structure and function, and these organelles are where proteins may perform a variety of tasks. It was easy to see that DEPs were largely distributed in the cytoplasm and nucleus of cells by looking at the amount and distribution of DEPs in each organelle (shown in **Figure 6A**).

All DEPs were tagged with GO function using Blast2Go (https://www.blast2go.com/), which shows the top 20 GO items from DEPs amongst various groups. GO enrichment findings of DEPs were classified as molecular function (MF), biological process (BP), and cellular component (CC). We found that DEPs following primary and secondary stimulation were considerably enriched in defensive response, inflammatory response, and reactivity to external stimuli compared to the control group (shown in **Figure 6B**). Compared with the primary stimulation group, DEPs that appeared after secondary stimulation were enriched in response to interferon−beta and defense response (shown in **Figure 6B**).

Annotations for DEPs were made using the KEGG pathway database; they are presented in **Figure 6**. Compared with the control group, DEPs that were up-regulated after primary and secondary stimulation were significantly enriched in rheumatoid arthritis, diabetes mellitus, allograft rejection and inflammatory disease (shown in **Figure 6C**). Compared with the primary stimulation group, KEGG enriched Th1 and Th2 cell differentiation, antigen processing and presentation, Th17 cell differentiation and other immune processes in DEPs up-regulated in the secondary stimulation group (shown in **Figure 6C**).

Of note, the KEGG enrichment of DEPs in the three datasets all showed metabolic patterns. However, different from the first two datasets (**Figure 6C**. BvsA, CvsA), KEGG of up-regulated DEPs in the third datasets (**Figure 6C**. CvsB) was enriched in metabolic processes such as glycolysis/gluconeogenesis, pyruvate metabolism, nicotinate and nicotinamide metabolism, pentose phosphate pathway and so on.

### Correlation between proteomic and transcriptomic results

We counted the number of genes/proteins that were significantly up-regulated in BMDMs of MRL/lpr mice after secondary stimulation (ie, DEGs and DEPs that were up-regulated in all three datasets above), and found 5 molecules (Calhm6, Isg15, Gbp2, Iigp1, Ifi47) tended to be up-regulated in both the transcriptome and proteome, while 1 molecule (Samhd1) was down-regulated in proteome and up-regulated in transcriptome, and their details are shown in **Table 7**.

**Table 7:** Gene/protein information showing correlation on transcriptome and proteome.

Gene Name	mRNA	Protein	Function
Calhm6	Upregulated	Upregulated	Also known as family with sequence similarity 26, member F (FAM26F), it was identified as a TLR signaling-derived membrane molecule that plays an important role in various immune responses. The expression of FAM26F has been shown to be altered in a wide range of viral, bacterial, and parasite infections, pathophysiological illnesses, and cancers, according to several research (44).
Isg15	Upregulated	Upregulated	As shown in **Table 3**.
Gbp2	Upregulated	Upregulated	One of the interferon-inducible GTPases superfamily, it plays a key role in innate immunity against viral illness, and has broad antiviral characteristics that are vital for protecting immunity versus microbial and viral pathogens (45).
Iigp1	Upregulated	Upregulated	IIGP1 is a mouse-specific ISG belonging to the family of immune-related GTPases (IRGs), which is mainly induced by IFN-γ and plays an important role in protecting the host from viral infections and bacteria (46).
Ifi47	Upregulated	Upregulated	Ifi47 is an immune response gene that contributes to defense against bacteria and protozoa (47).
Samhd1	Upregulated	Downregulated	Also known as dendritic cell-derived IFN-γ-inducing protein (48). Antiviral and apoptosis responses are triggered by SAMHD1’s role in promoting the development of a complex seen between reverse transcriptase intermediate (RTI) and STING (49).

## Discussion

Recent scientific research has demonstrated the existence of memory in innate immunity of immune cells to “remember” a previous infection or injury (such as infection or vaccination) and thus demonstrate a higher response when challenged again by the same or even an unrelated pathogen (13). Studies have now found that the rheumatic arthritis, hypertension, and Alzheimer’s disease are all linked to innate immune memory (14–17). It has opened up new possibilities for studying host defenses and the pathophysiology of immune-mediated disorders thanks to an improved knowledge of innate immunity. In SLE, the generation of large autoantibodies, widespread deposition of immunological complexes, and aberrant adaptive and innate immune responses are hallmarks (5). Some studies have reported that SLE is also related to innate immune memory (21–23), but its specific mechanism needs further research. At present, it has been found that there are multiple gene expressions related to SLE in the sle-infected tissues, B cells, T - lymphocytes, myeloid cells and the peripheral circulation (50). A wide range of molecular and immune mechanisms play a role in SLE and point to significant Interferon characteristics. Notably, ISGs involved in IFN signaling, production and response were significantly overexpressed in SLE patients, such as IFN regulatory factors, STAT4, IFIH1, OPN, etc (51). After an antigenic challenge, it has been demonstrated that BMDM can be reprogrammed to acquire memory-like features, enhancing or suppressing the ensuing immune response, that is innate immune memory (52, 53). Furthermore, it has been shown that BMDM innate immune memory contributes significantly to disease (54, 55). We speculated that there were memory ISGs in BMDMs from lupus-prone MRL/lpr mice.

We first selected BMDMs from mice aged 16-18 weeks, but the trend of gene expression did not change significantly after two stimulations. We think that the immune system of the diseased mice has been disturbed, the innate immune memory is influenced by a variety of factors, so the upward trend in ISG expression during secondary stimulation is unstable. Subsequently, experiments were carried out using younger mice, and a stable trend was observed in MRL/lpr mice aged 10-12 weeks. Moreover, we also found that in ICR mice, ISG expression on BMDMs was higher after secondary stimulation, regardless of the age of the mice. Compared with the result that the expression of ISG in 16-week-old MRL/lpr lupus mice did not significantly increase, it also shows that when the immune system is out of whack, the innate immune memory in lupus mice causes an ambiguous rise or fall in ISG levels. BMDMs were then subsequently extracted from MRL/lpr mice aged 10-12 weeks. qRT-PCR analysis found that ISGs associated with SLE were highly expressed in pre-stimulated BMDMs and peaked at 4 h, indicating the presence of IFN memory. In previous reports, multiple viral infections were associated with early-onset SLE, and the patients were generally adolescents (56–58). Primary infections in childhood are usually asymptomatic, but the virus remains dormant in the cells of an affected individual and can be reactivated at any time, affecting the immune system (56). Our study also suggests that external stimuli, such as viral infection, are associated with early-onset SLE, and also confirm that the activation of innate immune memory may have a role in the development of SLE.

To further study the relationship between innate immune memory and SLE pathogenesis, we stimulated BMDMs of MRL/lpr mice once and twice with IFN-γ and LPS, respectively. Then RNA-seq technology was used to obtain DEGs, and we discovered that, when compared to unstimulated BMDMs, there were some common DEGs engaged in the process of histone methylation and acetylation at the time of primary and secondary stimulation. There are numerous ways that cells can undergo epigenetic alterations, but innate immune cells with memory mostly show these changes in chromatin conformation and histone modifications. Changes in histone methylation and acetylation play a major role in innate immune memory (59). After primary or secondary stimulation, we discovered that DEGs implicated in histone methylation and acetylation processes appeared in MRL/lpr murine BMDM. They included H3f3b, MeCP2, Setdb, and L3mbtl3, whose aberrant expression suggested that histone methylation took place in BMDM (60–62). Also, alterations in the expression of Hat1, Hdac, Hdac5, Hdac6, and Hdac10 suggest that histone acetylation may have taken place at this time (63). At the same time, after differential expression analysis, 191 genes were found to be up-regulated in both stimulations. MRL/lpr mice’s BMDMs exhibit a greater expression of some genes after a second stimulation, indicating that they have a strong memory. GO enrichment analysis of these genes revealed that they were associated with cellular responses to interferon and external stimuli, defense responses, and innate immune responses. KEGG enrichment showed that they were involved in disorders caused by a viral infection, NOD-like receptor signaling pathway, TNF signaling pathway, RIG-I-like receptor signaling pathway, Toll-like receptor signaling pathway, JAK-STAT signaling pathway. Macrophages are innate immune cells that express pattern recognition receptors (PRRs) that can recognize the structure of foreign antigens: pathogen-associated molecular patterns (PAMPs) and endogenous danger signals: damage-associated molecular patterns (DAMPs), and produce corresponding ‘s answer (64). PRRs include Toll-like receptors (TLRs), RIG-I-like receptors (RLRs), Nod-like receptors (NLRs), Hin-200 family proteins and intracellular DNA receptors (65). Previous reports suggest that PRRs are involved in immune memory responses (66, 67). Our study found that after the second stimulation of BMDMs, the NOD-like receptor signaling pathway, RIG-I-like receptor signaling pathway, and Toll-like receptor signaling pathway had higher expression of SLE-related genes. It is shown that its related receptors have memory ability, which strengthens the recognition of pathogens, supporting the previous research results. Next, we screened out 12 genes with more obvious PPI interactions: Irf7, Stat1, Rsad2, Eif2ak2, Usp18, Isg15, Ddx58, Ifit1, Uba7, Ifit3, Ifi44, Ifih1. All of them were IFN-induced genes. Most of these memory ISGs have been shown to play an important role in the onset and progression of SLE (shown in **Table 2**). There are few reports on SLE of Uba7, but it may be coupled with ISG15 to play a role together. It has been reported that the expression of Isg15 is relatively high in patients with SLE and correlates with disease activity before treatment (68). Therefore, we identified them as candidate key genes on the innate immune memory pathway affecting SLE. Faster and higher expression of memory ISGs upon secondary stimulation is supported by our data.

Subsequently, a proteome study was carried out, and it was shown that 10 DEPs were up-regulated and 20 were down-regulated. Five of them (Isg15, Calhm6, Gbp2, Iigp1, Ifi47) showed the same trend of transcriptome change, and one (SAMHD1) had the opposite trend of transcriptome change, and they were related to the innate immunity of host defense against viruses and bacteria (shown in **Table 7**). DEPs were mostly located in the nucleus and cytoplasm, manifest in the process of innate immunity, and were involved in autoimmune and autoinflammatory diseases. The synthesis of proteins requires multiple post-translational modifications, and the modified proteins can change in structure and function, so there is no specific correlation between the expression levels of gene mRNAs and their corresponding proteins (69, 70). Despite the fact that protein as well as transcript levels of biomolecule expression did not show a strong correlation, we could see that after the second stimulation, both the transcriptome and proteome showed higher expression than the first stimulation of cytokines, demonstrating that IFN stimulation produces innate immune memory in MRL/lpr mouse BMDMs. Notably, we found that SAMHD1 was up-regulated in the transcriptome and down-regulated in the proteome when BMDMs were stimulated for a second time. In cells were transfected with SAMHD1 and infected with HP-PRRSV, the transcription of ISG15 as well as ISG56 was observed to be considerably enhanced (71). It suggested that enhancement of SAMHD1 may be associated with the initiation of innate immunity against viral infection. According to research, SAMHD1’s expression is controlled by many factors such as methylation, acetylation, and phosphorylation (72), furthermore, the phosphorylation study of SAMHD1 demonstrates that the upregulation SAMHD1 protein is largely in an unphosphorylated condition (73). It is known that protein synthesis requires post-translational modifications such as phosphorylation, glycosylation, small ubiquitin-related modifiers, and proteolytic cleavage. The modified proteins can change in structure and function. Therefore, SAMHD1 is up-regulated in the transcriptome, while the expression in the proteome may show a downward trend (70, 73). Studies have reported that SAMHD1 mutations are highly correlated with SLE, and SLE patients often have SAMHD1 gene deletions (74). DNA fragments are liberated from stalled forks to accumulate in the cytoplasm of cells underexpressing SAMHD1, activating the cGAS-STING pathway and inducing the production of pro-inflammatory type I IFN (75). We found that SAMHD1 protein was significantly down-regulated in pre-stimulated BMDMs, and down-regulation of SAMHD1 promoted ISG release, which also verified that memory ISG was highly expressed after the second stimulation. These ISGs work together in SLE patients to aggravate the occurrence and development of the disease.

More importantly, GO and KEGG analysis of up-regulated proteins in BMDMs after the second stimulation showed that they were enriched in metabolic patterns such as glucose metabolism. The occurrence of innate immune memory is known to be closely related to cellular metabolic regulation and epigenetics (76), which involve many central cellular routes of metabolism such as glycolysis, oxidative phosphorylation (OXPHOS), glutamate lysis, and fatty acid as well as cholesterol syntheses. Studies have shown increased rates of glycolysis in pro-inflammatory macrophages. Glucose-derived pyruvate is transformed into lactate following macrophage stimulation, which is then discharged from the cell (77). Because of this metabolic adaptability, innate immune cells can provide the efficient energy and building blocks needed for activation in a timely manner. Increased glycolysis is common in macrophages with different training stimuli such as beta glucan, BCG, and oxLDL. According to an increasing body of data, high glycolytic metabolic rates can control histone methylation and acetylation, forming the metabolic basis of innate immune memory (78). Several metabolites produced by glycolysis have been shown to be cofactors for DNA and histone methyltransferases, demethylases, as well as deacetylases and histone acetyltransferases (79). The metabolism and epigenetic reprogramming were the main mechanisms of innate immunological memory. The two, however, work together to tightly combine, coordinate, and orderly control the process, and the metabolites support the activity of histone-modifying enzymes by acting as substrates or coenzymes. The studies mentioned above proved that epigenetic changes such histone acetylation and methylation may take place when innate immune cells created innate immune memory. They cooperate with other processes to preserve innate immune memory traces that enable cells to react more quickly and forcefully when stimulated in the future.

## Conclusion

In summary, in MRL/lpr mice: 1) the expression of PRRs in innate immune cells was altered, and the expression increased after acquiring the memory phenotype to enhance the recognition of pathogens. 2) Enhanced inflammatory response, innate immune cells release a large number of ISGs through IFN-related pathways to promote the secondary response. 3) The related metabolic patterns of innate immune memory cells are enhanced, such as glucose metabolism, to adapt to changes in cellular response patterns. 4) Epigenetic reprogramming may have occurred, affecting histone modifications in downstream signaling pathways to generate innate immune memory. Therefore, multiple mechanisms work together to generate innate immune memory and affect the occurrence, maintenance and progression of SLE. During the pathogenic stage of SLE, gene and/or environmental factors, such as viral infection, “initially stimulate” macrophages to initiate disease. During the developmental stage of SLE, previously stimulated and trained macrophages are more likely to activate and activate PRRs, thereby inducing the activation of IFN-related pathways. Our study found that when the IFN pathway is activated again, ISGs will produce faster and higher expression. At this time, the enhancement of metabolic methods such as glycolysis also enhances the innate immune cells’ capacity to react to secondary stimuli and release pro-inflammatory factors. This is where enhanced tissue damage is triggered under chronic inflammatory conditions.

In order to better understand how SLE is induced and progressed, we conducted an in-depth study of the mechanism of innate immune memory in BMDM and its impact on SLE. It also sheds light on the disease’s pathophysiology, opening the door to novel and more effective ways to detect and treat SLE at an early stage.

## Data availability statement

The original contributions presented in the study are publicly available. This data can be found here: National Center for Biotechnology Information (NCBI) Sequence Read Archive (SRA) (accession no. PRJNA888089).

## Ethics statement

The animal study was reviewed and approved by Committee on the Ethics of Animal Experiments of Zhejiang Chinese Medical University Zhejiang Chinese Medical University.

## Author contributions

RQL and KO designed research; KC, DW, RL performed the experiments; TW performed experimental guidance; KC, XS and TZ analyzed data; KC wrote the paper; RQL revised the paper and acquired funding; KO gave critical advice. All authors contributed to the article and approved the submitted version.

## Funding

This study is supported by 2021 Annual Scientific Research Fund of Zhejiang Chinese Medical University (No. 2021JKGJYY028), the National Natural Science Foundation of China (No. 81673863, 82104798), the intermural program of NICHD, NIH, ZIA HD001310-34.

## Acknowledgments

We appreciate the experimental support from the Public Platform of Medical Research Center, Academy of Chinese Medical Science, Zhejiang Chinese Medical University and National Institute of Child Health and Human Development(NICHD), NIH.

## Conflict of interest

The authors declare that the research was conducted in the absence of any commercial or financial relationships that could be construed as a potential conflict of interest.

The reviewer LL declared a shared parent affiliation with the authors KC, DW, RL, XS, TZ and RL to the handling editor at the time of review.

## Publisher’s note

All claims expressed in this article are solely those of the authors and do not necessarily represent those of their affiliated organizations, or those of the publisher, the editors and the reviewers. Any product that may be evaluated in this article, or claim that may be made by its manufacturer, is not guaranteed or endorsed by the publisher.
